# Supplementary material for: The Cdc14 Phosphatase Controls Resolution of Recombination Intermediates and Crossover Formation during Meiosis
Source: Int J Mol Sci. 2021 Sep 10;22(18):9811. doi: 10.3390/ijms22189811 (PMC8470964; doi:10.3390/ijms22189811)
Supplement: Supplementary file 1 [file ijms-22-09811-s001.zip › ijms-1368721-supplementary.pdf]

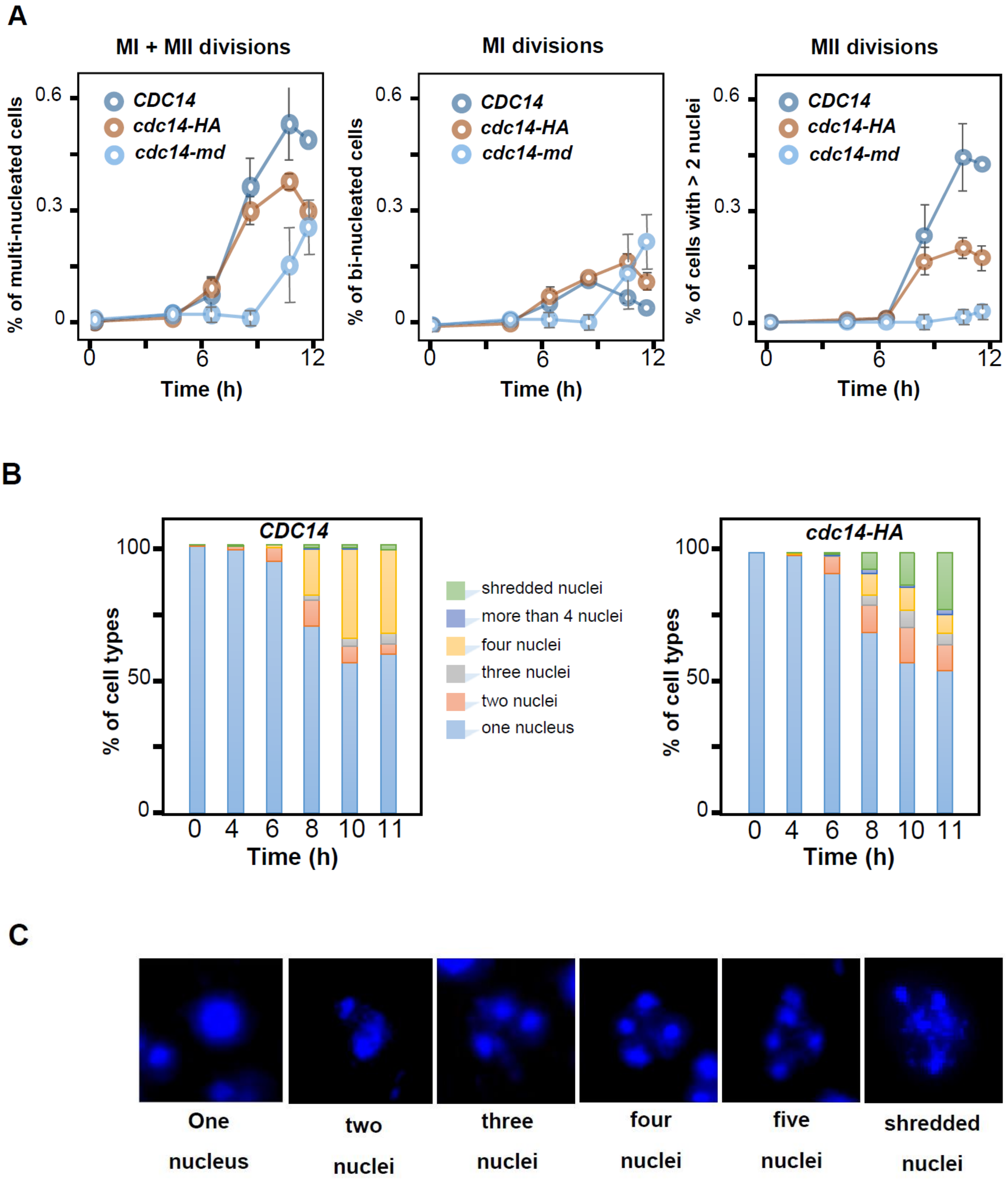

Figure S1. Cdc14 is required during meiosis for correct nuclear division. (**A**) *cdc14-HA* cells (JCY844) sustain both nuclear divisions although with subtly slower kinetics than wild type cells (JCY840) predominantly during the second round of nuclear segregation. *cdc14-md* cells (JCY2376) do not undergo the second nuclear division (**B**) Quantification of the number of nuclei as well as other aberrant nuclear structures during meiosis in *CDC14* (JCY840) and *cdc14-HA* (JCY844) cells. (**C**) Representative images of all nuclear structures quantified in (B).

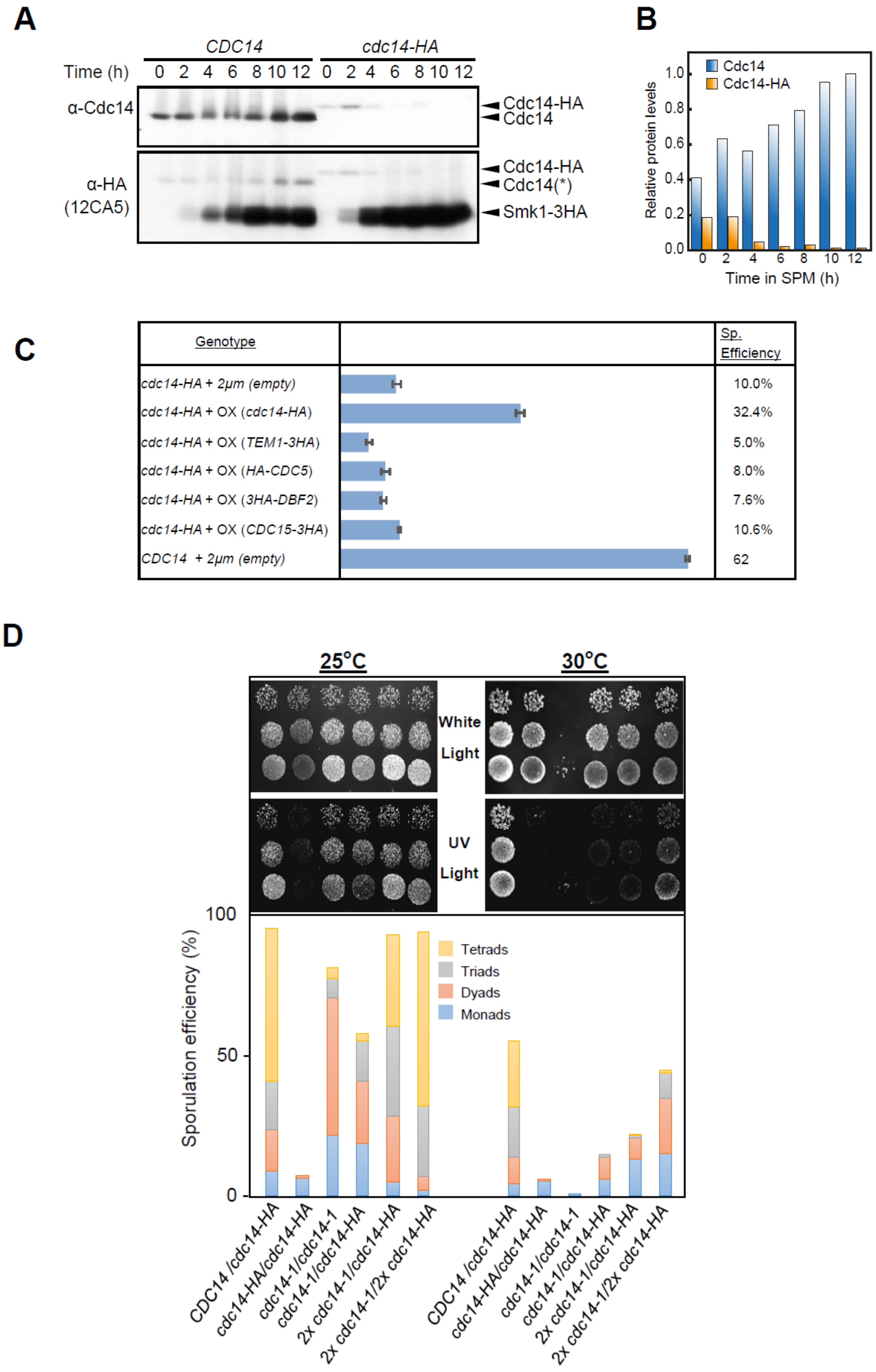
A
CDC14
cdc14-HA
Time (h)
0 2 4 6 8 10 12 0 2 4 6 8 10 12
α-Cdc14
α-HA
(12CA5)
Cdc14-HA
Cdc14
Cdc14-HA
Cdc14(*)
Smk1-3HA
B
Cdc14
Cdc14-HA
Relative protein levels
0.0 0.2 0.4 0.6 0.8 1.0
0 2 4 6 8 10 12
Time in SPM (h)
C
Genotype
Sp. Efficiency
cdc14-HA + 2μm (empty) 10.0%
cdc14-HA + OX (cdc14-HA) 32.4%
cdc14-HA + OX (TEM1-3HA) 5.0%
cdc14-HA + OX (HA-CDC5) 8.0%
cdc14-HA + OX (3HA-DBF2) 7.6%
cdc14-HA + OX (CDC15-3HA) 10.6%
CDC14 + 2μm (empty) 62
D
25°C
30°C
White
Light
UV
Light
Sporulation efficiency (%)
0 50 100
Tetrads
Triads
Dyads
Monads
CDC14 /cdc14-HA
cdc14-HA/cdc14-HA
cdc14-1/cdc14-1
cdc14-1/cdc14-HA
2x cdc14-1/cdc14-HA
2x cdc14-1/2x cdc14-HA
CDC14 /cdc14-HA
cdc14-HA/cdc14-HA
cdc14-1/cdc14-1
cdc14-1/cdc14-HA
2x cdc14-1/cdc14-HA
2x cdc14-1/2x cdc14-HA

Figure S2. Reduced levels of Cdc14 causes the sporulation defect observed in the *cdc14-HA* mutant. (**A**) Analysis of Cdc14 protein levels in wild-type (JCY902), and *cdc14-HA* (JCY904) cells. Top panel shows immunodetection using a polyclonal α-Cdc14 (yE-17) antibody. The same blot was reanalyzed using α-HA (12CA5) (bottom panel). (*) denotes residual signal from the earlier α-Cdc14 detection. *SMK1-3HA* is also expressed in both *CDC14* and *cdc14-HA* strains. (**B**) Quantification of Cdc14 protein levels shown in (B). Values were normalized against the maximum signal corresponding to the 12 h time-point in the wild type (**C**) Sporulation efficiency of *cdc14-HA* diploids transformed with multi-copy plasmids carrying MEN genes (GGY102/GGY103/GGY104/GGY105) and *cdc14-HA* (GGY93). Only overproduction of the Cdc14 phosphatase rescues the sporulation defect. (**D**) Dityrosine autofluorescence of different mutant combinations as well as the control strains grown and sporulated on plates at 25 ◦C and 30 ◦C. *cdc14-1* homozygous diploids (JCY2353) sporulate at high efficiency under semi-permissive temperature forming preferentially tetrads whereas *cdc14-HA* homozygous diploids do not sporulate at any temperature (JCY840). Combinations, and variable copy number, of the mutant genes can rescue the sporulation defect at different degrees (JCY2365/JCY2354/JCY2356).

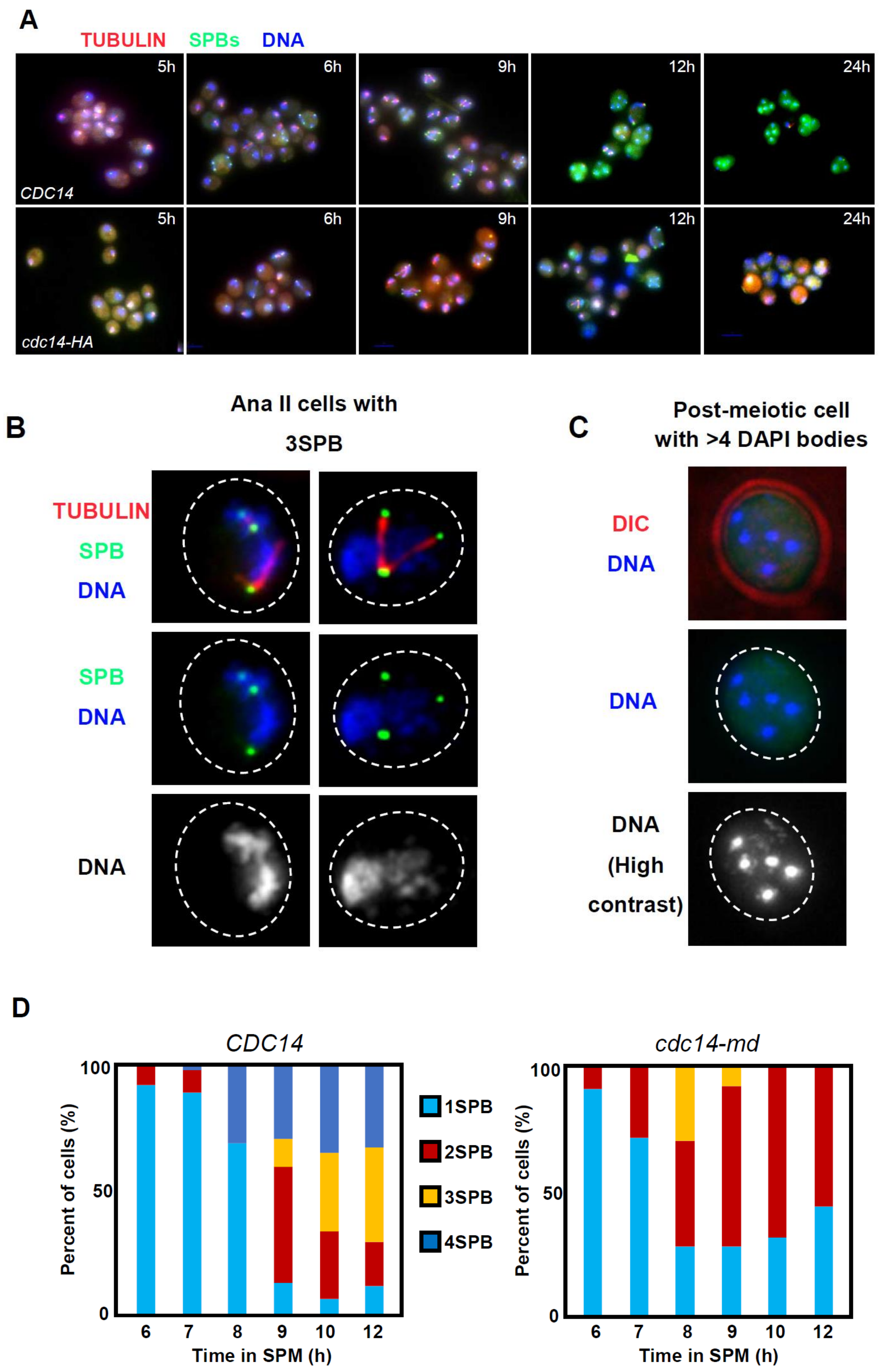
A
TUBULIN SPBs DNA
5h
6h
9h
12h
24h
CDC14
cdc14-HA
B
Ana II cells with 3SPB
TUBULIN
SPB
DNA
SPB
DNA
DNA
C
Post-meiotic cell with >4 DAPI bodies
DIC
DNA
DNA
DNA (High contrast)
D
CDC14
cdc14-md
Percent of cells (%)
100
50
0
6 7 8 9 10 12
Time in SPM (h)
1SPB
2SPB
3SPB
4SPB

Figure S3. Meiotic *cdc14* mutant cells re-duplicate their SPBs and assemble meiosis I and meiosis II spindles. (**A**) Representative images of fixed cells at different time-points during synchronous parallel time courses for both *CDC14* (JCY892) and *cdc14* mutant (JCY893) cells. *cdc14-HA* cells initiate both meiotic divisions, as visualized by different fluorescence markers, but they do not complete sporulation. (**B**) Some *cdc14-HA* (JCY893) cells display abnormal number of SPBs and atypical spindle conformations. (**C**) Example of the terminal phenotype of a post-meiotic *cdc14-HA* (JCY893) cell. (**D**) *cdc14-md* cells display strong SPB re-duplication defects and arrest with two nuclei.

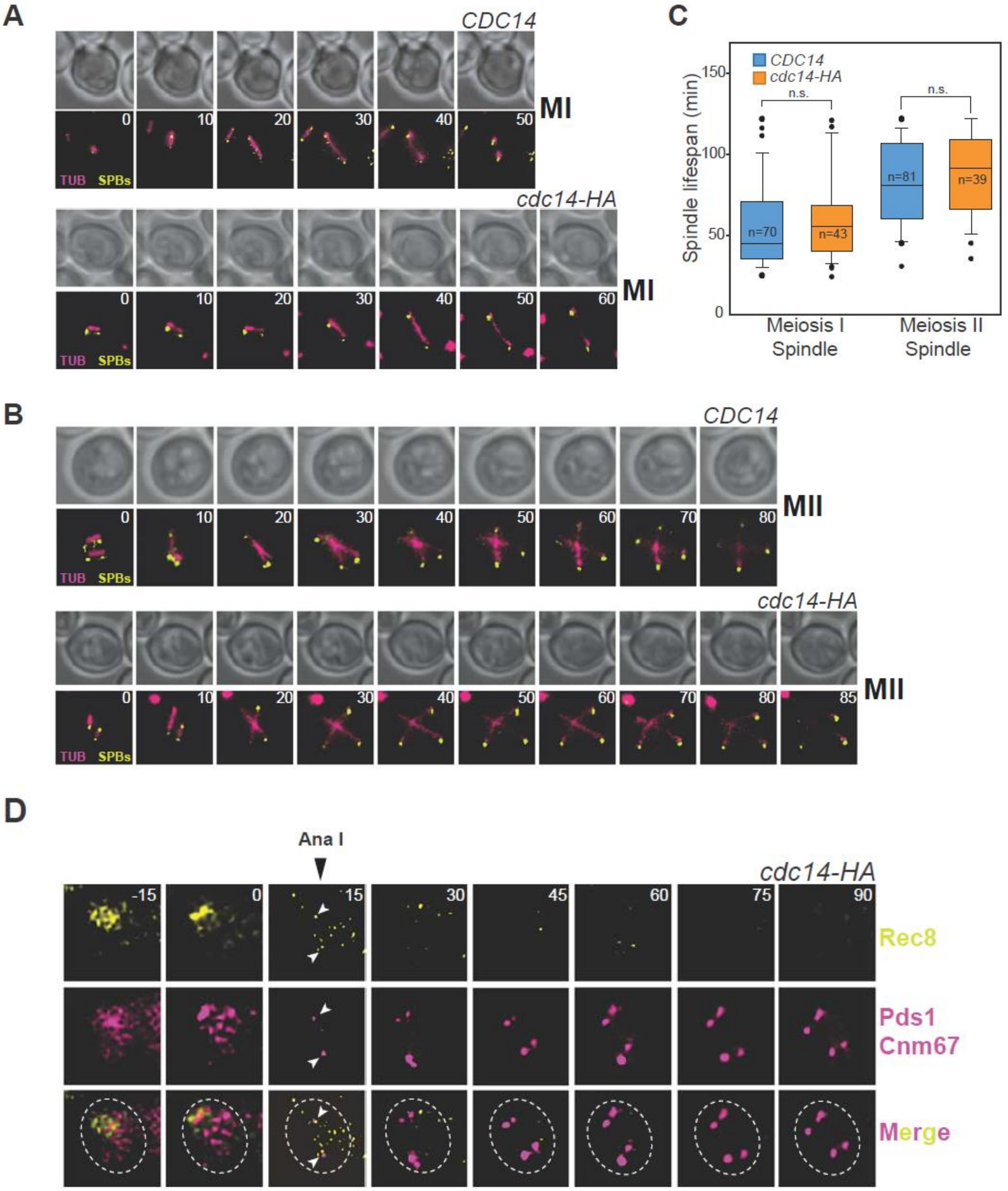

Figure S4. Meiotic spindle lifespan and dynamics are not altered in the meiosis-deficient *cdc14-HA* mutant**.** (**A**) Live-imaging of wild-type and *cdc14-HA* cells (GGY53 and GGY54, respectively) undergoing the first meiotic division carrying GFP-tubulin (red) and Spc29-CFP (green). (**B**) Visualization of spindle and SPB dynamics in the same strains (GGY53 and GGY54) undergoing the second meiotic division. For both A and B, microscopy fields from at least four different positions and from two separate wells were analyzed. A minimum of three time-courses per strain were run, acquired images were processed and movies generated (Video S1 and S2). Representative cells are shown. (**C**) Quantification of spindle lifespan from cells undergoing MI or MII. Meiotic deficient *cdc14-HA* cells (GGY54) show similar spindle dynamics compared to the wild type when completing both meiotic divisions. Box plots depict median number of spindle lifespan with whiskers representing upper and lower 1.5 interquartile range. Black dots represent outliers. Statistical test was performed using a twotailed unpaired *t*-test. (**D**) Example of a meiotic *cdc14-HA* (JCY2404) cell transiting from prophase I to complete both meiotic divisions. Rec8-GFP (yellow) and Pds1-tdTomato/Cnm67-tdTomato (magenta).Time 0 (min) was considered the last frame where Pds1 was visualized and SPBs stayed in close association. Data obtained from Video S3 and S4.

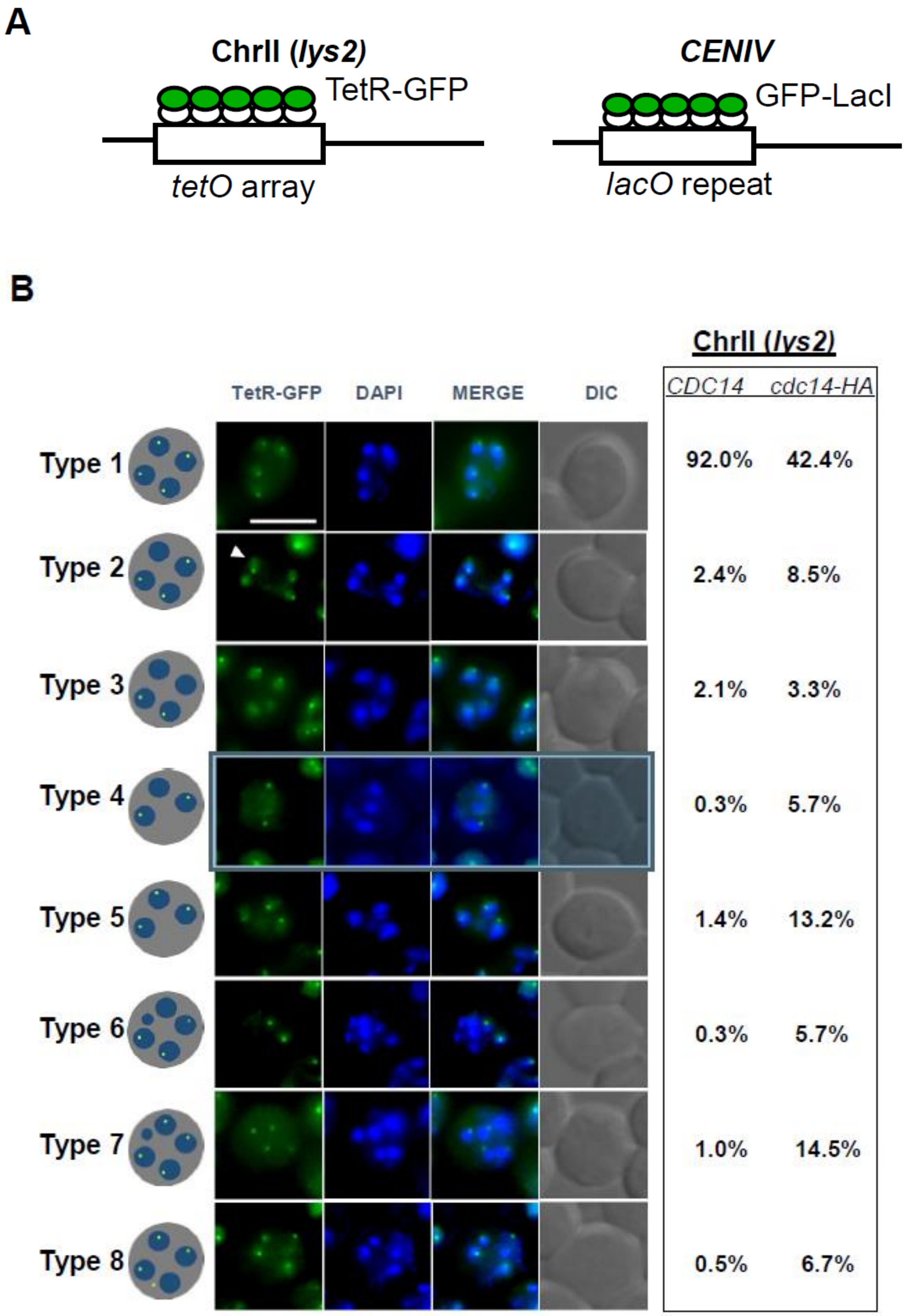

Figure S5. Lack of Cdc14 in meiosis causes missegregation of chromosomes at both meiotic

divisions. (**A**) Scheme depicting the chromosome GFP-tagging system placed at an interstitial *locus*
within chromosome II (left) and at a centromere proximal *locus* within chromosome IV (right). (**B**)
Representative images and frequencies of the observed types of distribution of interstitial chromosome II GFP dots in homozygosis of both *cdc14-HA* mutant (JCY2230) and wild-type (JCY2231) meiotic cells. Arrowheads indicate diffuse GFP in the nucleus. For chromosome segregation fidelity analysis (Fig 3D), different percentages from depicted were calculated from considering only cells with four nuclei (type 1, 2 and 3). . Cells were fixed in formaldehyde and nuclei stained with DAPI. Bar=5µm.

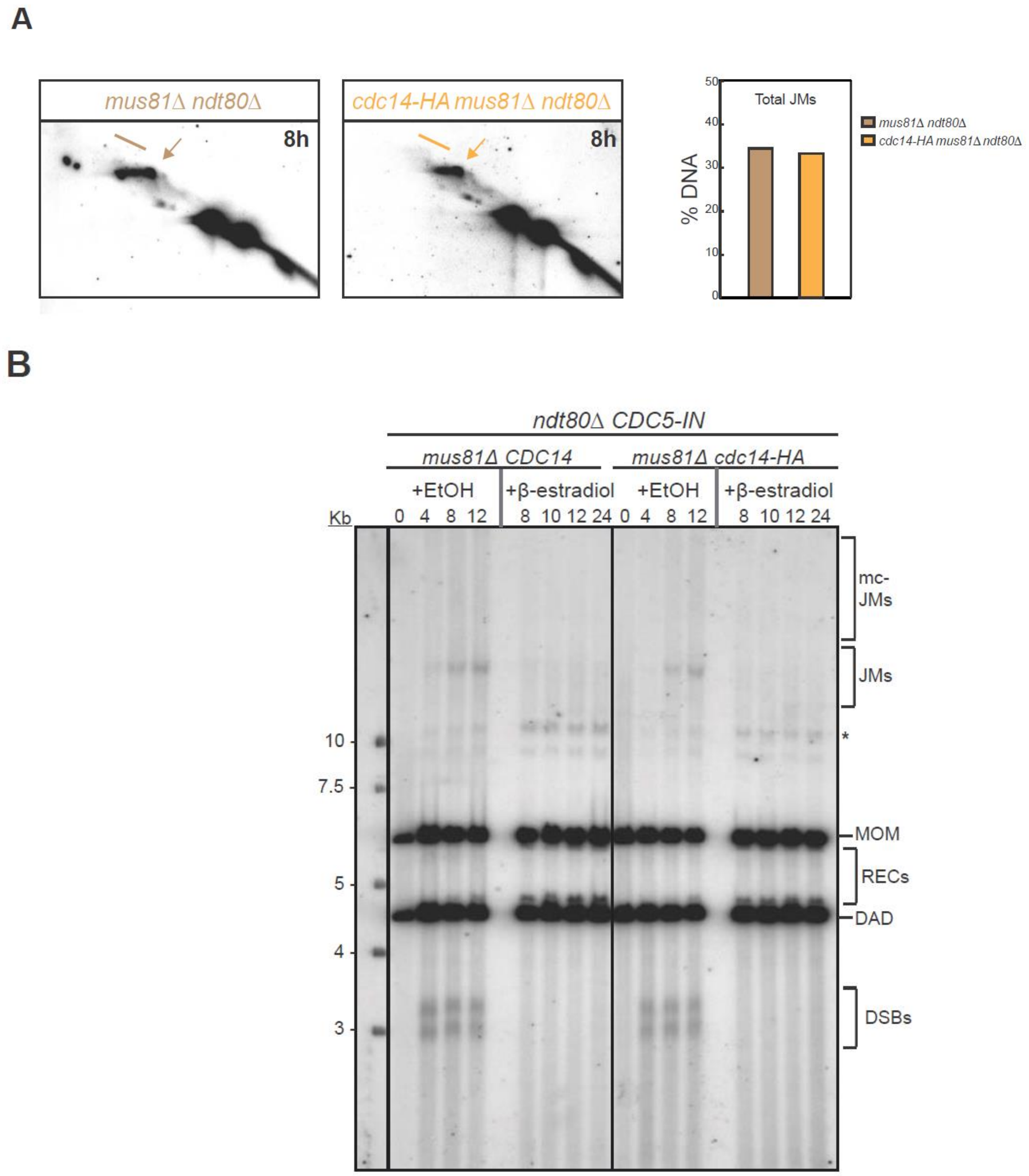

Figure S6. Accumulation of JMs is analogous in *cdc14-HA* in the presence or absence of Mus81. (**A; left**) Representative 2D gels showing recombination intermediates in *mus81*

ndt80, and *mus81 cdc14-HA ndt80* prophase-arrested cells 8 h after meiotic induction. (**A; right**) Quantification of DNA species from (2D-gels). (**B**) Comparison of JM abundance and resolution from the Southern blot shown in (Fig 5A). Asterisk indicates meiosis-specific non-characterized recombination products.

Table S1. *S. cerevisiae* strains used in this study. SK1 background strains were used throughout the study, unless otherwise specified.

Strain	Genotype
JCY840	*MAT a/α ho::LYS2/", lys2/", ura3/", leu2::hisG/", trp1::hisG/", arg4-nsp/", his4x/"*
JCY844	*MAT a/α ho::LYS2/", lys2/", ura3/", leu2::hisG/", trp1::hisG/", arg4-nsp/", his4x/", cdc14-3HA::KanMX4/"*
GGY92	*MAT a/α ho::hisG/LYS2, lys2, ura3(Dsma-pst::hisG)/ura3, HIS4::LEU2-(NBam;ori)/his4x, Zip1-GFP(at AA700)/", leu2::hisG/", cdc14-3HA::KanMX4/CDC14*
GGY91	*MAT a/α ho::hisG/LYS2, lys2, ura3(Dsma-pst::hisG)/ura3, HIS4::LEU2-(NBam;ori)/his4X or LEU2(NgomIV;ori)-URA3/his4x, Zip1-GFP(at AA700), leu2::hisG/", cdc14-3HA::KanMX4/"*
JCY902	*MAT a/α ho::hisG/", ura3/", leu2::hisG/+, lys2/", trp1/", SPC29-CFP::TRP1/", DON1-dGFP-KanMX/", pSMK1-3HA-URA3*
JCY904	As JCY902 but *cdc14-3HA::KanMX4/"*
GGY53	*MAT a/α ho::hisG/", ura3/", leu2::hisG/+, lys2/", trp1/", SPC29-CFP::TRP1/", TUB-dGFP-URA3/"*
GGY54	*MAT a/α ho::hisG/", ura3/", leu2::hisG/+, lys2/", trp1/", SPC29-CFP::TRP1/", TUB-dGFP-URA3/", cdc14-3HA::KanMX4/"*
JCY2196	*MAT a/α ho::hisG/", lys2/", ura3/", trp1/", his4/", leu2::hisG/", cdc14-3HA::KanMX4/"*
PRY151	*MAT a/α ho::LYS2/", lys2/", ura3/", leu2::hisG/", trp1::hisG/", spo11-Y135F-HA::URA3/", pCLB2::3HA-CDC14::KanMX4/"*
JCY2247	As JCY2235 but *cdc14-3HA::KanMX4/"*
JCY2270	*MAT a/α ho::LYS2/", lys2/", leu2::hisG/", ura3/", arg4-nsp/", trp1::hisG/", his4x/", spo11-Y135F-HA::URA3/"*
JCY2280	As JCY2270 but *cdc14-3HA::KanMX4/"*
JCY2232	*MAT a/α ho::hisG/", lys2/", ura3(Δsma-pst::hisG)/", leu2::hisG/", his4X::LEU2-(NgoMIV;ori)-URA3/HIS4::LEU2-(NBam;ori)*
JCY2231	As JCY2232 but *cdc14-3HA::KanMX4/"*
YJM7692	*MAT a/α ho::LYS2/" ura3/" leu2::hisG/" trp1::hisG/" his3::hisG/" YEN1-myc9::URA3/"*
YML7693	As YML3952 but *cdc14-3HA::KanMX4/"*

JCY2164	*MAT a/α ho::LYS2/", lys2/", ura3/", leu2::hisG/", trp1/", his4x::LEU2-URA3/", YEN1-9A-myc18::URA3/", cdc14-3HA::KanMX4/", ZIP1-eGFP(at AA700)/"*
GGY102	JCY844 with pSJ103 (*CDC15HA3-URA3)*
GGY93	JCY844 with pPD.2 (*CDC14HA3-URA3)*
GGY103	*JCY844 with pSJ57 (HA3-DBF2-URA3)*
GGY104	JCY844 with pSJ56 (*TEM1HA3-URA3*)
GGY105	JCY844 with pSJ29 (*HACDC5-URA3*)
JCY2353	*MAT a/α ho::LYS2/", lys2/", his4x/", leu2::hisG/", ura3/", cdc14Δ::KanMX4/", trp1::cdc14-1::TRP1::LEU2/"*
JCY2365	*MAT a/α ho::LYS2/", lys2/", his4x/", leu2::hisG/", ura3/", cdc14Δ::KanMX4/cdc14-3HA::KanMX4, trp1::cdc14-1::TRP1::LEU2/trp1 arg4-nsp/ARG4*
JCY2354	*MAT a/α ho::LYS2/", lys2/", his4x/", leu2::hisG/", ura3/", cdc14-3HA::KanMX4/cdc14Δ::KanMX4, trp1::cdc14-1::TRP1::LEU2/"*
JCY2356	*MAT a/α ho::LYS2/", lys2/", his4x/", leu2::hisG/", ura3/", cdc14-3HA::KanMX4/cdc14-3HA::KanMX4, trp1::cdc14-1::TRP1::LEU2/"*
JCY2222	*MAT a/α ho::LYS2/", lys2/", ura3/", leu2::hisG/", his4/", Cyc1p-LacI-GFP-URA3/", trp1::LacO-TRP1 (at CENIV)/"*
JCY2224	As JCY2222 but *cdc14-3HA::KanMX4/"*
JCY2284	*MAT a/α ho::LYS2/", lys2/", ura3/", leu2::hisG/", his4/", Cyc1p-LacI-GFP-URA3/ura3, trp1::LacO-TRP1 (at CENIV)/trp1*
JCY2286	As JCY2284 but *cdc14-3HA::KanMX4/"*
JCY2327	*MATa/α ho::LYS2/" his4/" ura3::Cyc1p-LacI-GFP-URA3/" trp1::LacO-TRP1/" CNM67-mCherry::natMX4/CNM67*
JCY2326	*MATa/α ho::LYS2/" his4/" ura3::Cyc1p-LacI-GFP-URA3/" trp1::LacO-TRP1/" CNM67-mCherry::natMX4/CNM67*
JCY2251	*MAT a/α ho::LYS2/", his4/", trp1/", lys2::TetOx240:URA3/lys2, leu2::LEU2 tetR-GFP/leu2*
JCY2248	As JCY2251 but *cdc14-3HA::KanMX4/"*
JCY2331	*MAT a/α ho::LYS2/", his4/", trp1/", lys2::TetOx240:URA3/", leu2::LEU2 tetR-GFP/", CNM67-mCherry::natMX4/CNM67*
JCY2330	As JCY2331 but *cdc14-3HA::KanMX4/"*

PRY53	*MAT a/α ho::LYS2/" leu::hisG/" spo11-Y135F-HA::URA3/",TUB-dGFP-URA3/" SPC29-CFP::TRP1/" HIS4X/hisx4x*
PRY55	*MAT a/α ho::LYS2/" leu::hisG/" spo11-Y135F-HA::URA3/" TUB-dGFP-URA3/" SPC29-CFP::TRP1/" cdc14-3HA::KanMX4/" arg4/" HIS4/his4x*
PRY99	*MAT a/α ho::LYS2/" leu2/" his4x/" trp1-ura3/LacO-TRP1 (at CENIV))- Cyc1p-LacI-GFP-URA3, YEN1-9A/"*
PRY108	*MAT a/α ho::LYS2/" leu2/" his4x/" trp1-ura3/LacO-TRP1 (at CENIV)- Cyc1p-LacI-GFP-URA3 cdc14-3HA::KanMX4/" YEN1-9A/"*
PRY121	*MAT a/α ho::LYS/" leu2/" SPC29-CFP::TRP1/" YEN1-9A/" TUB-dGFP-URA3/" his3/", cdc14-3HA::KanMX/"*
PRY123	*MAT a/α ho::LYS/" leu2/" SPC29-CFP::TRP1/" YEN1-9A/" TUB-dGFP-URA3/" his3/"*
PRY132	*MAT a/α ho::LYS2/ho::hisG, lys2/" leu2::hisG/" TUB-dGFP-URA3/" SPC29-CFP::TRP1/", pCLB2:3HA-CDC14::KanMX4/", his3/"*
PRY182	*MATa/α ho:.LYS2/" his3 or his4/" leu2/" LacOTRP1 (at CENIV)-Cyc1p-LacI-GFP-URA3/" YEN1-9A/" lys2/" pCLB2::3HA-CDC14::KanMX4/"*
JCY2389	*MAT a/α ho::hisG/" lys2/" ura3(Δsma-pst::hisG)/" leu2::hisG/" his4X::LEU2-(NgoMIV;ori)-URA3/HIS4::LEU2-(NBam;ori) pCLB2::3HA-CDC14::KanMX4/"*
JCY2385	*MAT a/α ho::hisG/" lys2/" ura3(Δsma-pst::hisG)/" leu2::hisG/" his4X::LEU2-(NgoMIV;ori)-URA3/HIS4::LEU2-(NBam;ori) ndt80::hphMX6/" cdc14-3HA::KanMX4/"*
JCY2390	*MAT a/α ho::hisG/" lys2/" ura3(Δsma-pst::hisG)/" leu2::hisG/" his4X::LEU2-(NgoMIV;ori)-URA3/HIS4::LEU2-(NBam;ori) ndt80::hphMX6/"*
JCY2399	*MAT a/α ho::hisG/" lys2/" ura3(Δsma-pst::hisG)/" leu2::hisG/" his4X::LEU2-(NgoMIV;ori)-URA3/HIS4::LEU2-(NBam;ori) ndt80::hphMX6/" pCLB2::3HA-CDC14::KanMX4/"*
JCY2440	*MAT a/α ho::hisG/" lys2/" ura3(Δsma-pst::hisG)/" leu2::hisG/" his4X::LEU2-(NgoMIV;ori)-URA3/HIS4::LEU2-(NBam;ori) ndt80::hphMX6/" mus81::hphMX4/" cdc14-3HA::KanMX4/" pCDC5-Gal4-ER-pGAL1-CDC5-natMX4/"*
JCY2442	*MAT a/α ho::hisG/" lys2/" ura3(Δsma-pst::hisG)/" leu2::hisG/" his4X::LEU2-(NgoMIV;ori)-URA3/HIS4::LEU2-(NBam;ori) ndt80::hphMX6/" mus81::hphMX4/" pCDC5-Gal4-ER-pGAL1-CDC5-natMX4/"*
JCY2444	*MAT a/α ho::hisG/" lys2/" ura3(Δsma-pst::hisG)/" leu2::hisG/" his4X::LEU2-*

	(NgoMIV;ori)-URA3/HIS4::LEU2-(NBam;ori) mms4::hphMX6/" pCLB2::3HA-SGS1::KanMX4/"
JCY2446	As JCY2444 but *pCLB2::3HA-CDC14::KanMX4/"*
JCY2448	As JCY2444 but *yen1::natMX4/"*
JCY2529	*MATa/α ho::LYS2/" mms4Δ:.hphMX4/" HIS4B::LEU2/leu2 his4x::LEU2-URA3 pCLB2::3HA-CDC14::TRP1/ pCLB2::3HA-CDC14::KanMX4 trp1 pCLB2-3HA-CDC20 (KANMX6) sgs1::CLB2p-HA3-SGS1::KanMX5/" YEN1-9A-18MYC::URA3/YEN1 his3::hisG*
JCY2421	*MATa/α ho::LYS2/" ura3/" leu2::hisG/" trp1::hisG/" his3::hisG/" YEN1-9A-myc18::URA3/"*
JCY2376	*MATa/α ho::LYS2/" lys2/" ura3/" trp1/" his4/" leu2::hisG/" pCLB2::3HA-CDC14::KanMX4/"*
JCY2478	*MATa/α ho::LYS2/" lys2/" trp1/" HIS4B::LEU2/hix4x::LEU2-URA3 ura3/" yen1Δ::CNAT/" sgs1::CLB2p-HA3-SGS1::KanMX5/" mms4Δ:.hphMX4/" pCLB2-3HA-CDC20 (KANMX6)/"*
JCY2480	*MATa/α ho::LYS2/" ura3/" leu2::hisG/"trp1::hisG/" his3::hisG?/? sgs1::CLB2p-HA3-SGS1::NatMX4/sgs1::CLB2p-HA3-SGS1::KanMX ms4::hphMX4/" yen1::NAT/YEN1 pCLB2-HA3-CDC20::KanMX6/" HIS4(NewBamH)-LEU2/his4X-LEU2-URA3*
JCY2469	*MATa/α ho::LYS2/" lys2/" trp1/TRP1 his4x::LEU2-URA3/HIS4B::LEU2 ura3/" sgs1::CLB2p-HA3-SGS1::KanMX5/" mms4::hphMX4/MMS4 yen1Δ::NatMX4/"*
JCY2502	*MATa/α ho::LYS2" his4x::LEU2-URA3/HIS4B::LEU2 sgs1::CLB2p-HA3-SGS1::KanMX5/" pCLB2-3HA-CDC20 (KANMX6)/" pCLB2::3HA-CDC14::TRP1/" his3::hisG/? mms4Δ::hphMX4/MMS4*
JCY2508	*MATa/α ho::LYS2/" sgs1::CLB2p-HA3-SGS1::KanMX5/" HIS4B::LEU2/his4x::LEU2-URA3*
JCY2406	*MATa/α ho::LYS2/" ura3/" leu2::hisG/" trp1::hisG/" his3::hisG/" REC8-GFP::LEU2::KanMX4/", PDS1-tdTomato::KiTRP1/", CNM67-tdTomato::NatMX4/"*
JCY2404	As JCY2406 but *cdc14-3HA::KanMX4/"*
JCY892	*MATa/α ho::hisG/" ura3/" leu2::hisG/LEU2 lys2/" trp1/" SPC29-CFP::TRP1/" TUB-dGFP-URA3/"*
JCY893	*MATa/α ho::hisG/" ura3/" leu2::hisG/LEU2 lys2/" trp1/" SPC29-CFP::TRP1/" TUB-dGFP-URA3/" cdc14-3HA::KanMX4/"*

Video S1. Meiotic spindle lifespan and dynamics in *CDC14* cells. Live imaging of cells (GGY53)
undergoing both meiotic divisions carrying GFP-tubulin (red) and Spc29-CFP (green).

Video S2. Meiotic spindle lifespan and dynamics in *cdc14-HA* cells. Live imaging of cells (GGY54)
undergoing both meiotic divisions carrying GFP-tubulin (red) and Spc29-CFP (green).
Video S3. Kinetics of Rec8-GFP association/removal in *CDC14* cells. Sequence of Rec8-GFP wildtype
cells (JCY2406) at 15-minute intervals displaying kinetics of cohesin assembly and removal.
Video S4. Kinetics of Rec8-GFP association/removal in *cdc14-HA* cells. Sequence of Rec8-GFP
cdc14-HA cells (JCY2404) at 15 minutes interval displaying kinetics of cohesin removal.
